# Bert Vallee—A 20th Century Adventure(r) in Zincology

**DOI:** 10.3390/ijms222413393

**Published:** 2021-12-13

**Authors:** Claus Jacob, Ahmad Yaman Abdin, Frederieke Köhler, Wolfgang Maret

**Affiliations:** 1 Division of Bioorganic Chemistry, School of Pharmacy, Saarland University, D-66123 Saarbruecken, Germany; yaman.abdin@uni-saarland.de (A.Y.A.); shavit.koehler@gmail.com (F.K.); 2 CNRS, Centrale Lille, University Lille, UMR 8181–UCCS–Unité de Catalyse et Chimie du Solide, University Artois, F-59000 Lille, France; 3 Departments of Biochemistry and Nutritional Sciences, School of Life Course and Population Sciences, Faculty of Life, Sciences and Medicine, King’s College London, Franklin-Wilkins Bldg, 150 Stamford St., London SE1 9NH, UK

**Prelude**

Bert Lester Vallee (1919–2010) has been among the most important biochemists of the 20th century, a pioneer in metalloproteins and discoverer of numerous zinc proteins and enzymes, such as carboxypeptidase, alcohol dehydrogenases and metallothioneins. His scientific achievements are condensed in over 600 publications, and articles relying on and citing his research are suited to fill entire bookshelves. Although Bert Vallee, as a scientist, has left a significant legacy on science, his more personal side and encounters have mostly escaped public observation. We deem this oversight rather unfortunate, as his personality, and indeed personal circumstances, have been truly turbulent and must have influenced his scientific career, from his birth as Bertold Blumenthal in the small village of Hemer in post-World War I Germany via Switzerland to New York and then Boston. Together with public records, the less obvious attributes and actions recommend a more holistic biography. On the occasion of Bert Vallee’s 100th birthday in 2019, we have attempted to provide such an inclusive and rounded résumé. We also propose that a similar rounded approach will add additional layers to the biographies of contemporary scientists, considering social, economic, political, and historical environments and their mutual interactions, which tend to shape the scientist embedded in them.

## 1. Introduction

This Special Issue on “Thiophilic Metals: An ancient love for sulfur at the heart of biochemistry” has been compiled in celebration of the centenary of Bert Lester Vallee (1919–2010), a founding father and pioneer of zinc biochemistry, whose 100th birthday would have been on the 1st of June 2019. Bert L. Vallee is renowned in science as an adherent investigator of numerous zinc proteins and enzymes and, together with his student Marvin Margoshes, counts as the discoverer of the zinc-sulfur protein Metallothionein (MT) [1]. It is therefore appropriate to mark this event with a suitable biography of Bert Vallee as a scientist, a colourful person, and a seafarer of the turbulent 20th century.

**Figure 1:**
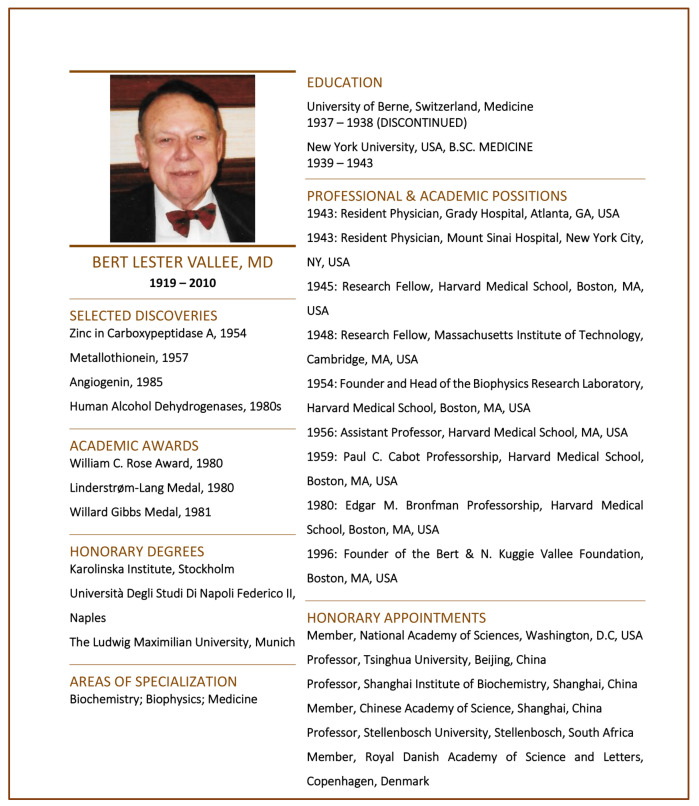
Example of a more traditional tabular CV, in this case summarizing the scientific career and main scientific achievements of Bert Lester Vallee on a single page.

In our opinion, the biography of an eminent scientist such as Bert Vallee should not simply be a tabular collection of dates indicating when and where the person has graduated from, has been appointed to academic positions or indeed has made certain discoveries or published specific manuscripts (Figure 1). Regrettably, many short biographies of colleagues past and present resemble such basic and sterile résumés, similar to a rollcall of events and achievements, which is very common inside the community of natural scientists. Indeed, a tabular résumé serves its purpose, for instance, when applying for positions, grants, and awards, or dealing with other purely scientific affairs. Yet, there is always more to scientists, and their personal side ought not to be ignored. Scientists in the laboratory are not detached from the persons they were just a minute ago outside the building. They cannot be reduced just to their scientific output. Scientists are complex individuals embedded within the equally complex historical, political, social, and economic environment of their day.

We therefore opted in the following sections to construct an extended résumé model, against which biographies of great scientists will count for the contextual richness of their time and place. In the following Section 2, we shall present our model which will account for social, economic, political, and historical environments, turning the traditional *curriculum vitae* into a nested *curriculum professionis*. In Section 3, we tell the story of Bert Lester Vallee aka Bertold Blumenthal. Section 4 is dedicated to the brilliant scientific career of Bert Vallee, and in Section 5 we conclude.

## 2. From a Restricted Résumé to a Rounded Description of the Scientist

Traditional scientific biographies regularly turn a blind eye to the different layers of the persons and/or indeed their interactions with the environment. Such an unfortunate approach is sterile to the context and environment of the scientists and the reciprocal interplay which may have influenced their scientific performance, almost as if personal issues behind the scientific output are irrelevant and should not be discussed [2,3,4,5,6]. This is rather curious, as modern historians take a more comprehensive view on historical figures, from politics to the arts. It would almost be impossible, for instance, to comprehend the artistic output of Vincent van Gogh without considering his very personal idiosyncrasies and penchants.

**Figure 2:**
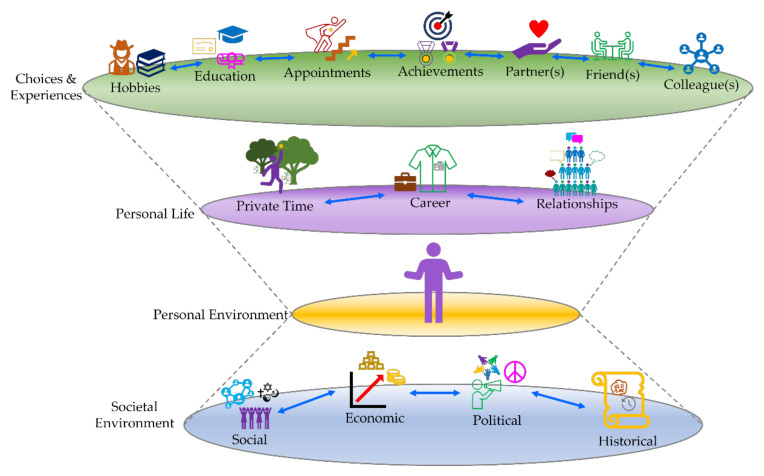
The placing-on-stage (mise-en-scène) of the individual. The bottom layer represents the Societal Environment, which is a result of the interaction of social, economic, political, and historical factors. The next layer, Personal Environment, is the societal environment filtered by the personal particularities of the actor. The Personal Life layer depicts the outcomes of the actor engaging with her/his personal environment distinguished by the different roles one plays. The upper layer, Choices and Experiences, are moments that shape us and make us who we are. There is a feedback mechanism between the different layers of the model represented by the dotted grey lines.

We therefore propose that the same rounded and holistic approach should also be applied to scientists according to the layered scheme shown in Figure 2. Such a rounded biography should, for instance, take note of and account for the historical circumstances under which scientists were active, their personal situation within these environments, and how these aspects have then intermingled with their scientific activity to produce the scientific output we cherish today. This approach would provide contrast to the choices they made along their paths and ensuing consequences, put beautifully by Goethe “Der Handelnde ist immer gewissenlos” (the doer is always unscrupulous). Indeed, although scientific output is often the predominant part of such biographies, it is only the part visible to the world, the tip of the iceberg of a more general approach.

Interestingly, such a rounded approach of intentionally considering the person behind the scientist discloses additional sources and paves the way of presenting the biographies, for instance by sampling and citing from private letters, in which the persons themselves describe major events, or quoting public documents and petitions kept in local archives and the testimony of contemporaries. This, in turn, provides us with much more detail and a better conception of the historical situation when compared to a traditional tabular résumé. Such historical sources are often unavailable or ignored, yet they provide a valuable addition to the bare numbers and facts as they describe events from an autobiographic angle, in the own words of the originator’s wit and personal assessment. These sources give a voice to the colleagues who speak in their own words, echoing, for example, from old letters.

## 3. To Capture the Early Bert—A Layered Model (of) Biography

This manuscript provides new insights into the life of Bert Lester Vallee. The assembly has only been possible through the help and support of his relatives and friends who agreed to share unpublished photos, letters, and documents from which this story is told. We only quote three letters from 1948, 1958 and 1960 and cite others when necessary. We shall illustrate it in our layered model, providing a holistic biography by discussing the journey of Bert Vallee from pre-World War II Germany to the United States (U.S.). Indeed, the biography of Bert Vallee, which spans a large swathe of the 20th and early 21st century, is perfectly suited to allow himself to tell us his story, in pictures and words. In his biography, which, as he himself would say in a moment of great excitement, is as hot as a “two barrel shotgun”, we shall combine information on his career, appointments, research interests, and achievements with his personal interests and journey from his birthplace, the German town of Hemer, to Boston, Massachusetts—a journey which, in turn, was embedded in and driven by the turbulences of the history of the 20th century. We propose that this approach, as shown in Figure 2, is superior to traditional biographies, and indeed the only way to grasp the forces behind the science and the scientist. In a metaphorical way, to fully capture Bert Vallee, you must first capture Bertold Blumenthal.

### 3.1. Bertold Blumenthal

**Figure 3:**
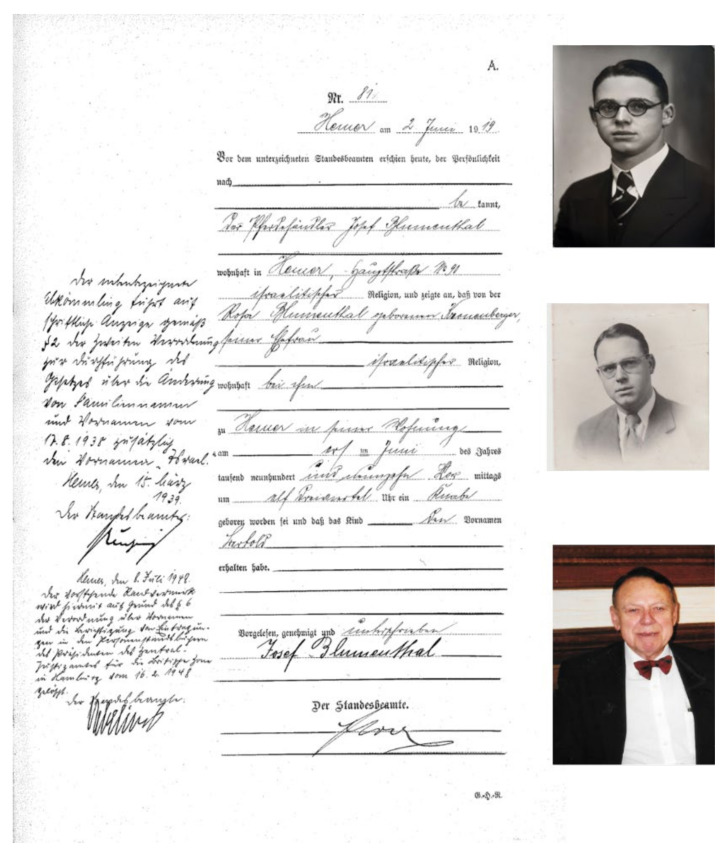
Photos of the birth certificate of Bert L. Vallee issued in 1919 by the city of Hemer in Germany and of the young Bertold and the old(er) Bert. (Copy of the birth certificate was kindly provided by Mr. Thomas Eberhard from the city archive of the town of Hemer, Germany. The photos on the side of the certificate were provided by the relatives mentioned in the acknowledgments).

To begin with, Bert Vallee had not always been called Bert Vallee, and, similar to his metaphorical shotgun, his life also has two barrels to consider. His biography is that of Bertold Blumenthal, a bright young student with a promising future in early 20th century Germany, studying medicine at the University of Berne in Switzerland, and of Bert Vallee, the famous American biochemist of the 1950s and decades to follow (Figure 3 birth certificate and photos young and old(er)).

Bert Vallee was born as Bertold Blumenthal in the small town of Hemer, not far from Iserlohn, in what is today the German State of North Rhine Westphalia, on the 1st of June 1919, just four weeks before the signing of the Treaty of Versailles, which officially marked the end of the First World War (Figure 3, birth certificate). His father, Josef Blumenthal (1882–1958), was a descendent of a respected family of horse traders, whose long and fruitful history in Hemer can be traced to the 18th century, together with their stud farm and many relatives in town. His mother, Rosa, née Kronenberger, was not from Hemer. She was born in 1879 in the small village of Hoppstädten near Birkenfeld, today in the State of Rhineland-Palatinate. Besides Bertold, the couple had another son, Kurt Ziegbert, born in 1913 and father of a niece, Yona Kates, in Israel, and a nephew, Richard Bert Vallee, a professor at Columbia University in New York City. Bertold spent his youth in Hemer, attending school and subsequently the Gymnasium (High School or Secondary School) in nearby Iserlohn. The oral history of Hemer still bears memory of Bertold as a very intelligent and popular young boy.

Bertold obtained his degree for college admission, in Germany called Abitur, in Iserlohn in the summer of 1937 and moved to the University of Berne in Switzerland, where he matriculated on the 1st of November 1937 under the registration number 30993 to study medicine.

Up to this date, the biography of Bertold with Gymnasium, Abitur, and enrolment at university may be considered as common and not any different from what it would be today. Nonetheless, as discussed in our account, historical context must be considered in addition to personal and academic developments. Indeed, the moves of Bertold provide evidence of the closely intertwined interplay of the different layers.

### 3.2. A Sojourn from Germany to Switzerland and the United States

Here, we must turn our attention to politics. In the early 1930s, matters for the Blumenthal family had already turned sour as soon as the Nazi Party took over in Germany in January 1933, when Bertold was just 13 years old. Although the family continued to stay in Hemer, its members, as any Jews in the Third Reich, were exposed to suppression, discrimination and, later, pogroms. This also included the field of academia, where the number of Jewish students was grossly restricted and limited from 1933 and suffered de facto complete exclusion from higher education soon after the major racist laws were issued in 1935.

**Figure 4:**
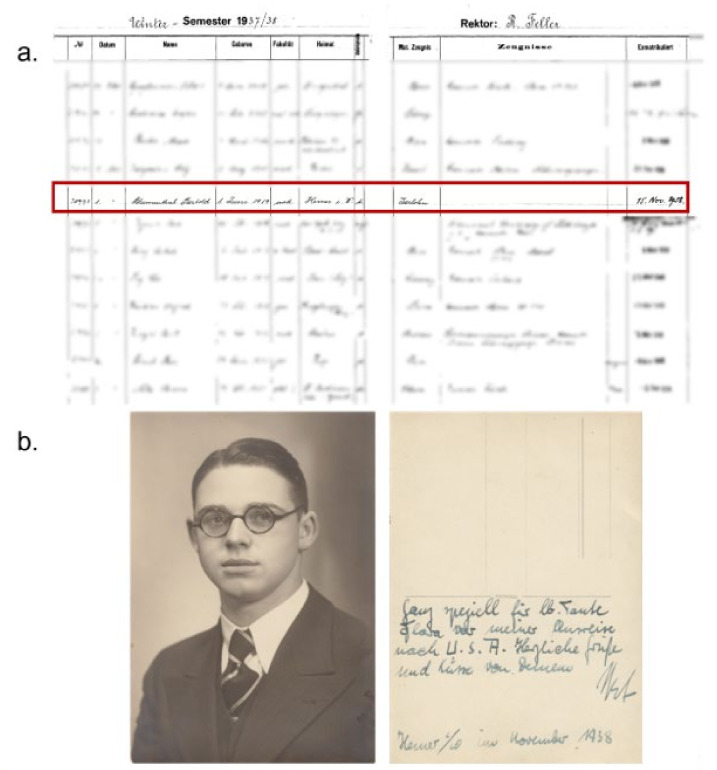
(**a**) Document from the University of Berne showing Bertold’s registration number, faculty, and ex matriculation date. Other names have been shaded purposively because of data protection concerns. (**b**) The postcard he sent from Hemer addressing his aunt Flora in November 1938 before his imminent departure to the U.S. (The registration document was kindly provided by Mr. Niklaus Bütikofer, university archive, University of Berne, Switzerland. The postcard was provided by the relatives mentioned in the acknowledgements).

Within this framework of political developments in the 1930s, it is therefore hardly surprising that Bertold decided to leave Germany and to move to Switzerland for his studies. The University of Berne is a German speaking university and studying there must not have been that different from taking a degree at a German university. Indeed, as the enrolment documents of the university from that year show, Berthold was not the only German student in his class, (Figure 4a).

In 1938, the political situation in Europe became increasingly tense, with Germany annexing Austria and large parts of Czechoslovakia. The suppression of Jewish citizens in the German Reich also escalated in the infamous Kristallnacht pogrom on the 9th of November 1938. It is therefore tragic, yet not surprising, that the personal situation of the Blumenthal family also changed. In that year, several members of the family decided to leave Germany and also Europe. Bertold’s older brother Kurt (1913–1961) was the first, and records show that he first went to Jerusalem, where he took up Palestinian citizenship, on the 18th of October 1938, as indicated on his German local registration card, before moving on and settling in the U.S.

On the 15th of November 1938, less than one week after the Kristallnacht pogrom, Bertold ex-matriculated from the University of Berne (Figure 4a). Just days later, on the 17th of November, he obtained a U.S. visa issued in the southern German city of Stuttgart, which he had probably already applied for earlier. Notably, during this turbulent month in 1938, he sent a postcard from Hemer with his photo and wrote “…very specially for my lovely aunt Flora before my departure to the US” (Figure 4b). Interestingly, we shall meet this postcard and photo again soon.

**Figure 5:**
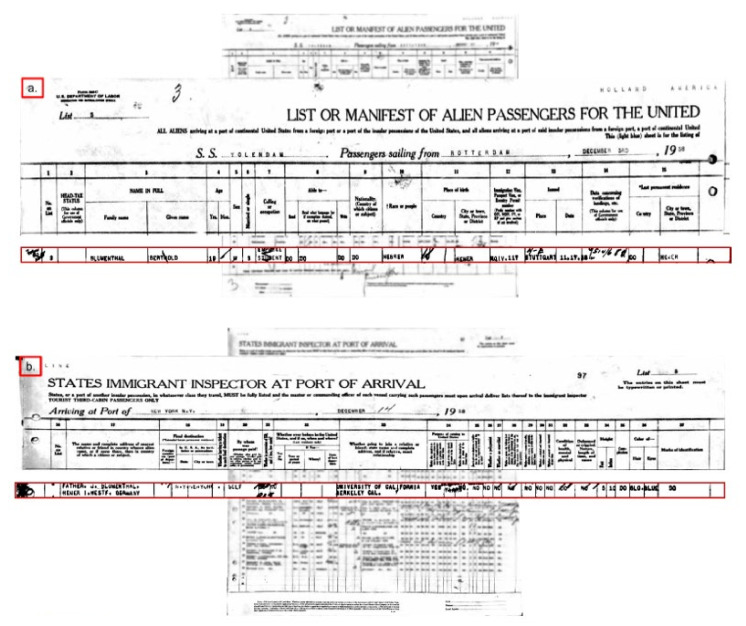
(**a**) boarding card and, (**b**) landing card of Bertold Blumenthal travelling from the Dutch city of Rotterdam to New York on board the SS Volendam in December 1938. Other names have been shaded purposively because of data protection concerns. (Photos adapted from www.ancestry.com, accessed on 8 December 2021).

Bertold was always quick in his decisions and even faster in his actions, and on the 3rd of December 1938, Bertold Blumenthal, as a student and official resident of Hemer, set sail from the Dutch harbour of Rotterdam onboard the SS Volendam, as passenger number 3 on list 3, to reach the New World, i.e., New York, on the 14th of December 1938 (Figure 5a. boarding card and, Figure 5b. landing card).

### 3.3. From Bertold to Bert Lester

On that notable day, the 14th of December 1938, Bertold Blumenthal, passenger 3 on list 3 according to his landing card and certified by the ship’s physician as in good health, 5 feet 10 inch tall, with blue eyes and blond hair, disembarked the SS Volendam as a student on his way to the University of California, Berkeley, corrected the spelling mistake in his first name Bertold and then decided to become Bert Lester Vallee, in his own words “…as you certainly know, Kurt and also myself have changed our names”.

Changing names means changing one’s self-perception and making a fresh start. Such a move is not entirely uncommon, especially when also changing countries, and, in the case of Bertold, also not entirely unexpected. Notably, the German word Blumenthal translates literally to *flower valley* in English and *vallée des fleurs* in French, a translation as part of Bert’s wider transformation once in the U.S. The new name also provides a clue to the French connection of the Blumenthal/Vallee family to French/German speaking countries such as Luxembourg and Belgium, where his father Josef spent some months after he left Germany as one of the last members of the family left in Hemer. Josef moved to Luxembourg on the 29th of June 1939, just weeks before the start of the Second World War, and then to the U.S., as did Bertold’s uncle Oscar (1899–1975), a horse trader in Germany who, according to a family member, subsequently entered the medical profession in the U.S.

Curiously, one of the first documents written by Bert Vallee in the U.S. is a postcard with his own photo, an exact copy of the one in Figure 4b, sent to Hemer to his cousin Karl Blumenthal in early 1939 “…as a souvenir and for remembrance”. This postcard is identical to the one sent to his aunt from Hemer. It is also written in German, yet dated in English, “New York City, February 9, 1939”, a clear indication of the transformation from Bertold to Bert. Indeed, Bert would continue to communicate with Karl for several decades, until the 1960s, usually in German, and, via these letters, we shall now allow him to tell us about his life in the U.S. during the 1940s and 50s in his own words.

### 3.4. The Early Years in the United States—From Blood to Zinc

“As you know, I have studied here medicine and obtained my Doctorate in 1943. I have subsequently been a resident physician in a large hospital in New York for a couple of years. In 1945, the government assigned me to a military research project. Since then I have been involved in research, which I am likely to continue. I do not practice medicine. In its place, I am employed at one of the best American universities in Boston, where I teach. My current position is similar to the one described as Private Docent in Germany. Nonetheless, it is not exactly the same, as the system here is totally different. I am planning to move to England next year (…)”. (Letter from a personal archive addressed by Bert to Karl and Brunhilde in Germany dated 16 July 1948, referred to as Letter 1948 thereafter).

Indeed, upon his arrival in the U.S., Bert had found the support of the mathematician Richard Courant (1888–1972) who served as his mentor during his studies in medicine at New York University [7]. It is often held that Bert was the one and only fellow of the Student Service of the League of Nations, although we could not find any relevant documentations on this statement. The League of Nations may be considered as a predecessor of the United Nations (UN). It was set up in early 1920 and dissolved on the 20 April 1946 after the foundation of the UN on the 26 June 1945. A stipend of the League of Nations for a student of German origin to study in the U.S. is rather interesting, as Germany had quit the League of Nations in 1933 and the U.S. did not even join the League of Nations in the first place [8].

League of Nations or not, Bert was always playing in a league of his own. After obtaining his MD, he interned as a resident physician at Grady Hospital in Atlanta and at Mount Sinai Hospital in New York City.

The United States had entered the Second World War after the Japanese Empire attacked Pearl Harbour in 1941, and the preservation of blood reserves for transfusions was a major challenge in the field and in hospitals around the world. In this situation, Bert and his skills were desperately needed, and in 1945, taking the challenge as a scientific opportunity, he assisted at Harvard Medical School (HMS) in the Blood Preservation Program of Edwin J. Cohn and John Edsall [7]. These colleagues were eminent scientists and among the founding fathers of the biophysical analysis of proteins. As part of the Blood Preservation Program, Bert was studying the viability of red blood cells by labelling them with radioactive iron, and soon tried to use zinc for the same purpose in leukocytes. Indeed, as Bert himself tells us in his letter from 1948, quoted before, his studies with red blood cells laid the foundation for his scientific career concerning the trace metal zinc.

It is dramatic and also curious that the upheavals of the 1930s and 1940s turned Bertold Blumenthal into Bert L. Vallee and towards zinc biology, and one may only guess what may have become of Bertold Blumenthal without the wider historical situation, an issue he himself reminisced about on several occasions, although “…it is not really the right place to discuss (….) and to investigate how it would have turned out, when this and that would have been done. It looks to me that it is too late for that now”, Letter 1948.

## 4. Metals in Biology

Indeed, the need for medical research during the Second World War had brought Bert to Boston, Massachusetts, where he turned to the determination of zinc in biological samples. Boston and zinc soon became his home, personally and scientifically. In Letter 1948, Bert tells his cousin Karl in Hemer “As you probably know, I am married to an American since last year. My wife also teaches at a university”. Bert had settled in Boston together with his wife Natalie Kugris (Kuggie) Vallee, whom he married in 1947 and who herself was an academic and served as a Professor of Biology at Lesley College, Cambridge, MA. On a personal note, he enjoyed driving an 8-cylinder Oldsmobile and a new Dodge and resided first at 81 Gainsborough Street, Boston in “…a furnished 3 bedroom flat, which adequately meets our purposes”, and later at 56 Browne Street in Brookline before moving to 300 Boylston Street, Boston for their later years.

Bert’s first publications stem from Mt Sinai Hospital, New York, NY in the late 1940s on reactive arthritis [9]. Once he had moved to Boston, he was immersed in his work, cherishing the holidays where he and his “…wife has had a little more leisure than usual as the university holidays have started”. Together, the academic couple clearly enjoyed Boston and, according to him, “…the perspectives for the next few years are excellent”, Letter 1948.

The next few years were indeed excellent, and the situation developed nicely for the Vallee couple in the 1950s, with Bert taking on prestigious positions at Harvard University and tackling important scientific problems in the field of trace element research, which resulted in numerous important discoveries and publications. In his own words:

“We have both a lot of work. You probably know that Kuggie teaches Biology at a college for girls. She is therefore very busy. Recently, I have been appointed as Extraordinary Professor in Medicine at Harvard University. As one consequence my workload has also increased, the usual consequence of such honours”. The quotation is from a handwritten letter of a personal archive and addressed by Bert to Karl and Brunhilde in Germany in 1960, referred to as Letter 1960 hereafter.

Indeed, Bert Vallee was appointed as Assistant Professor at Harvard Medical School in 1956, Paul Cabot Professor in 1959, and Senior Edgar Bronfman Professor in 1980. He was also elected as a Member of the National Academy of Sciences in 1974.

In Boston in the early 1940s, working together with John R. Loofbourow from the Massachusetts Institute of Technology (MIT), Department of Biology, Spectroscopy Laboratory, Bert applied emission spectroscopy with a direct current (DC) arc to biology, specifically to determine the metal content of blood cells. His focus on metal analyses continued for many years, as increasing the detection limit was critical for advancing our understanding of metals in biology. In 1954, Bert founded the Biophysics Research Laboratory at Peter Bent Brigham Hospital, now Brigham and Women’s Hospital.

For functional studies of the proteins and metalloproteins he began to isolate, many new instruments were employed and acquired once the first prototypes came on the market, e.g., for analytical ultracentrifugation, optical rotary dispersion, and circular dichroism (CD), including MCD, electron paramagnetic resonance (EPR), and rapid-scanning stopped-flow spectrophotometry. Bert focused on one element in his blood cell work, namely zinc. Subsequently, several breakthroughs followed, highlighting the association of zinc with enzymes and proteins. Bert Vallee worked with Hans Neurath from the University of Washington in Seattle to isolate the second known zinc enzyme, carboxypeptidase, in 1954 (after zinc was discovered in carbonic anhydrase by Thaddeus Mann and David Keilin in 1939) [10]. He also isolated alcohol dehydrogenase, and established the widespread use of this element in enzymatic catalysis [11,12]. As zinc is spectroscopically silent for most techniques, Bert developed methods to characterize the active sites of zinc enzymes by using cobalt as a substitute for zinc. Zinc biochemistry remained one of his chief interests in the field of inorganic biochemistry, and his comprehensive review on zinc physiology and biochemistry in 1993 remains a key reference, even today, with 3500 citations as of 20 August 2021 on Google Scholar [13].

### 4.1. The Center for Biochemical and Biophysical Sciences and Medicine (CBBSM)

During the first years of his studies and scientific career, Bert was affected and driven by political developments, first in Europe and then also in the U.S., a situation he acknowledges in his letters. In subsequent years, another external societal factor became a similarly important driving force in his career, namely economics. Today, outside funding from agencies or companies at universities is commonplace. In the 1950s, striking substantial financial deals with industry had been highly unconventional, something frowned upon, and only attempted by persons thinking outside the box. In his own words, Bert Vallee has been such a “Querdenker”, a lateral thinker able to identify and then also follow avenues off the trodden path and not immediately apparent to his contemporaries.

Indeed, Bert forged large alliances with private donors. He was successful in persuading the giant American agrochemical and agricultural biotechnology company Monsanto into backing his research on angiogenesis. This particular research grant from the 1970s added to millions of U.S. dollars, led to a major expansion in terms of facilities and equipment, and eventually, in 1985, cumulated in the isolation, characterisation, and sequencing of the first human organogenic factor, the blood vessel-inducing, tumour-derived protein angiogenin [14,15,16]. Moreover, thanks to substantial funding from the Samuel Bronfman Foundation, the Endowment for Research in Human Biology was established. The initial funding was tax-free, topped over USD 5 million, and was allocated to investigate the more practical applications of zinc, for example in human alcohol dehydrogenases, and possible links to the treatment of alcoholism. In the subsequent years, another USD 10 million were granted, eventually necessitating the expansion of Bert’s laboratory, which changed its name from the Biophysics Research Laboratory into the Centre for Biochemical and Biophysical Sciences and Medicine (CBBSM), with laboratories at Harvard Medical School and the Brigham and Women’s Hospital [7]. The name reflects the wide interests of Bert in the biomedical sciences. It would be safe to say that Bert Vallee had a vision, was a first-class negotiator, and profoundly understood the role finance played as a driver of science and scientific discovery.

In his well-equipped laboratory, Bert focused on a biochemistry component, i.e., enzyme mechanisms and zinc biology, a biophysics component, i.e., spectroscopy and kinetics, and a medicine component which was a reminder of his training as a physician. The overarching goals of his research were ensuring that it was translational, perhaps as a homage to belonging to a medical school, Harvard Medical School.

In the 1980s, the centre employed 70 staff. It had all the necessary facilities to be self-sufficient, namely instrumentation for metal analyses and biophysical measurements, protein and nucleic acid sequencing, cell culture and animal facilities, and a library with a significant number of key journals. The staff structure included other professors at the three academic levels, all under his tutelage, almost exclusively postdoctoral scientists from all over the world, technicians, administrators, and secretaries. The expansion of the work established him in many additional areas, such as alcohol research, specifically the enzymology of alcohol and aldehyde dehydrogenases, the molecular and kinetic mechanisms of zinc proteases, in particular carboxypeptidase, and developmental biology with the description of angiogenin and the physiological function of zinc in cell biology. Highlights in the order of these areas include the isolation and characterization of the multiple members of the human alcohol dehydrogenase family, made possible by the invention of a ternary complex affinity chromatography; the isolation of an aldehyde dehydrogenase inhibitor from a traditional Chinese medicine, suppressing alcohol drinking in animal models; the isolation of angiogenin, triggering blood vessel formation (angiogenesis), which turned out to be a ribonuclease and expanded work into the enzymology of these enzymes and the molecular *characterization of the placental ribonuclease inhibitor; zinc biology by using the model organisms Euglena gracilis* and *Xenopus laevis*; and showing the essential role of zinc in cell proliferation and in the cell cycle. Credit is due not only to the permanent staff in his laboratories, but also to the many people who started their careers in Boston under his aegis as a gifted science educator. The colleagues are too numerous to be named, yet clearly made seminal contributions.

### 4.2. The Discovery of Metallothionein

During his exploration of zinc proteins, there was a time in the 1950s when the question lingered whether cadmium, the congener of zinc in the same group in the periodic table of the elements, had a role in biology as it was found in many biological systems. Bert isolated a cadmium protein from horse kidneys, which had the highest amount of cadmium of several organs screened [1]. As it contained a relatively high amount of sulfur (Greek θεῖον theîon = “sulfur”) and several metal ions in addition to cadmium, it was named metallothionein. The discovery turned out to be somewhat of a Danaergeschenk (double-edged gift). The protein was refractory to giving away its function. Its role in zinc, copper, and cadmium metabolism continued to occupy the minds of scientists for the next sixty years. We now record 20,000 references on the protein. The topic of metallothionein is introduced here with reference to the focus of this Special Issue on metal–sulfur interactions, as metallothionein is an archetypal protein with such bonds. Coordination environments in proteins were poorly characterized in these early days and metallothionein is perhaps the first example with coordination of cysteine sulfur to cadmium and zinc in biology. Characterization of coordination of sulfur to copper and iron (and other metals) in proteins and interactions with methionine were discovered by others in the following years. Metal–sulfur interactions were also investigated in Bert Vallee’s laboratories with regard to zinc/sulfur coordination in enzymes, e.g., alcohol dehydrogenases, the inhibition of cysteines in proteins by metals ions such as silver and mercurials and vice versa the inhibition of zinc in active sites by sulfur-containing compounds, e.g., hypertensive drugs such as captopril, binding with their mercapto group to angiotensin-converting enzymes. (Editorial note-copied from wiktionary: The term mercaptan was introduced in 1832 by William Christopher Zeise (1789–1847) and is derived from the Latin mercurium captāns (capturing mercury) as the thiolate group bonds very strongly with mercury compounds.) The significance of sulfur binding to zinc has occupied Vallee for a long time. The topic was taken up over 40 years after the discovery of metallothionein by showing that the sulfur donor ligand confers redox activity on zinc, thus coupling zinc/thiolate coordination environments to redox signalling and providing a means of releasing very tightly bound zinc from proteins and by demonstrating a role of selenium biology in this process by utilizing the redox relationship between selenium and sulfur [17,18].

### 4.3. The Special Issue on Special Thiophilic Metals

Indeed, the coordination of so-called thiophilic metal ions to biological sulfur motifs, such as inorganic sulphide (S^2−^) ions or organic cysteine or methionine residues in proteins, provides a fertile ground for research in a specific field, which, nowadays, is often referred to as Metallomics [19]. It is therefore more than appropriate to commemorate the 100th birthday anniversary of Bert Vallee with a Special Issue on “Thiophilic Metals: An Ancient Love for Sulfur at the Heart of Biochemistry”.

In his own words, Bert was a Querdenker (lateral thinker), an expression borrowed from German. Today, the term has a certain negative connotation. Back in the mid to late 20th century, when Bert was at the peak of his academic career, the term was coined and attributed to him by his colleagues who knew him for his rigorous questioning of assumptions. This sceptical attitude towards assumptions was sometimes fruitful, leading to valuable and perplexing breakthroughs, among them the discovery of the metallothionein proteins.

In this Special Issue, Abdin et al. therefore revisit the discovery of metallothionein (MT) from the angle of philosophy of science to provide a clear historical and contemporary summary of research conducted on MT by Bert Vallee and his colleagues [20]. In their manuscript, the authors from natural sciences and philosophy join forces to analyse the research conducted on MTs. They also discuss new research strategies worthy of pursuit given the status quo. The authors also highlight the uniqueness of the discovery of MTs, which seems to have been upward-looking, exploratory, and utilizing mere interactions. Historically, the research conducted by Bert Vallee on the MT proteins has therefore been opposed to the predominant approaches in biological research of the day which have been mostly downward looking, hypothesis-testing, and utilizing interventions.

In their manuscript, the authors not only try to disambiguate a 60 year-old question about the function of MTs [20], they also pose the question whether traditional mechanistic explanations are sufficient or indeed exclusive in modern science. Alternative explanations, such as teleological explanations dealing with a specific function or purpose of a protein within a wider physiological context, may at first glance seem antiquated. Nonetheless, today they may represent valuable tools for simple(r) explanations in science and as proper trajectories to scientific investigations. In fact, most research into the MT proteins assumes the existence of an, albeit elusive, function, and that each protein serves a specific purpose. The studies of Bert Vallee and his followers in this field have therefore not only been mechanistic, they also show elements of teleology and personal intuition.

It is also interesting that the discovery of the MTs has been the result of a search for cadmium proteins rather than zinc proteins as a comprehensive review of the 60+ years of research on mammalian MTs summarizes [21]. There are quite a few instances where thiophilic metals in proteins and enzymes can exchange for each other depending on their specific binding constants. Later on, Bert Vallee would use this specific circumstance to label spectroscopically silent zinc proteins with cobalt and nickel ions [22].

A similar approach is taken by Catapano et al. in their contribution on the zinc transporter ZnT8, which is linked to the development of type 2 diabetes [23]. The authors begin their article by discussing the limitation of the model of the ZnT proteins based on homologous proteins of bacteria and the impact of structural differences on metal affinity and hence the role or function of the proteins. Here, the findings concur with the literature on the differences between the bacterial proteins and the human ZnT8 protein. The authors also noted that the C-terminal extension of the human protein with its three cysteine residues binds a zinc (II) ion. To study this further, nickel (II) ions in place of the zinc have been employed. The study also reports on the two most common variants of human ZnT8 with either tryptophan (W) or arginine at position 325 and the small differences in stability between the two variants. The authors discuss the involvement of the *C*-terminal cysteines and two histidines and another cysteine at the *N*-terminus of the ZnT8 molecule in a metal chelation and/or acquisition mechanism. This hypothesis is supported by recent high-resolution cryo-EM structural studies showing the coordination of these residues in the zinc binding site in the cytoplasmic domain. Together, this manuscript provides one of the first examples of metal-thiolate coordination in zinc transporters, a more recent aspect of metallomics that Bert Vallee would have certainly cherished.

Indeed, metal ions such as zinc do not roam freely inside organisms; inter- and intracellular metal trafficking is a tightly controlled logistical masterpiece, often performed by transport proteins and extraordinarily small concentrations of metal ions. Metal trafficking shuttles metal ions between metal transport proteins and storage sites and employs chaperones to insert metal ions into selected places of selected metallo-enzymes.

Bert Vallee witnessed the early days of this metallomics research at the turn of the millennium, although he could no longer actively contribute to it. In his honour, Hendricks et al. discuss the role and relevance of thiolate clusters of proteins in different taxonomic groups and report on such a controlled metal insertion procedure [24]. In their manuscript, they show the transfer of iron ions from the iron-sulfur cluster assembly proteins ISCA2 and ISCU to lipoyl synthase (LIAS) [24]. This enzyme is responsible for the biosynthesis of lipoic acid, which has several important biological roles. The biosynthetic step facilitated by LIAS is a double sulfur insertion to the precursor of lipoic acid.

Regarding metal trafficking, the researchers noted that ISCA2 transfers the Fe-S cluster most effectively and, therefore, is the likely physiological donor, with ISCU serving a redundant role as a back-up if required. Concurrent with previous findings, the manuscript suggests the formation of [4Fe–4S] clusters in downstream targets and discusses this process in the context of proteins where natural mutations have been implicated in patients with low levels of lipoic acid, resulting in severe disease states such as hyperglycemia. The authors also remark on an importance in the order of the cluster addition and propose that it is necessary for the auxiliary cluster site of LIAS to be occupied prior to reconstitution of the [4Fe–4S] cluster. In fact, this manuscript provides a salvo of sulfur and metal transport and cluster assembly activities in eukaryotes. In many ways, it continues the narrative which Bert Vallee started by discovering the first zinc–sulfur cluster proteins more than 60 years ago. LIAS also emphasize once more that metals in biology are not just interesting, they are also central to human health, an aspect always close to the heart of Bert Vallee MD.

From these manuscripts discussing the ways of discovery, metal transport, and cluster assembly, it is clear that the field(s) concerned with metals in biology have been constantly and steadily advancing in theory and also in practice since the beginning of the 20th century. The influence of Bert Vallee as one of the founding fathers of this modern field of research cannot and should not be underestimated; his scientific legacy is beyond any doubt. Amazingly, and as one may expect from Bert Vallee, he has also left the scientific community another rather unique legacy which we shall now discuss briefly before concluding.

### 4.4. The Vallee Foundation and Beyond

Bert and his wife Kuggie had no children. As a legacy, together they established the Bert L. and N. Kuggie Vallee Foundation (Valleefoundation.org) in 1996. It developed from the concept that short-term visits of university faculty abroad lead to very productive international interactions but are limited by a scarcity of funding mechanisms. Accordingly, Vallee Visiting Professorships (VVPs) were introduced, followed by symposia where research outcomes of the VVPs are discussed. The foundation has developed into a robust organization that supports originality, creativity, excellence, and leadership in biomedical research and education, helps young people in their career, and, in the spirit of N. Kuggie Vallee, encourages women in science. A biographical memoir, an introduction to a dedicated issue of the *Journal of Inorganic Biochemistry*, and an obituary summarize his achievements [7,25,26].

Bert seized every day, both personally and scientifically. One of his maxims he instilled in his students was: “You can always make up for lost money, but you can never make up for lost time”. As a true Querdenker of his days, he recognized the value of new scientific developments early and adjusted quickly to new playgrounds, moving on his research, never being static. He was a trendsetter in many areas which are now taken for granted and have acquired names much later, such as bottom-up research and translational research. Examples of dissemination of his work in the public domain, which the reader may find edifying and enjoyable, are his essays on alcohol in human history [27,28].

Bert worked until the last days of his life, already in his early nineties, and continued even after severe illnesses. He was an innovative medical educator and promoted academic medicine as a physician scientist. His legacy in the scientific literature comprises over 600 articles.

Bert was insightful, innovative, and forward looking. He never returned to Hemer as far as we know, and it appears to have been his plan all along; he writes “I have intentionally refrained from writing to anyone, and I would appreciate if you would not forward my address to anyone. Everyone who would then write to me may also simply not do it”. Letter 1948. Indeed, the last entry under his name in the book of Jewish citizens in Hemer is the day Bertold boarded the SS Volendam in December 1938. It is clear from his letters that Bert had moved on, and that he had left the old Bertold and Hemer behind.

Bert had always been a man of character and generosity; as a person he has been as colourful as a scientist, and as the numerous discoveries he accomplished over seven decades. He has been famous for his rather direct approach to science, and also to scientists. “Some learn it sooner, some later, and some simply not properly. It looks to me that the situation is not really amenable for a discussion of etiquette and good manners”, Letter 1948.

In Section 3.4, Bert’s testimony indicates a will to move to England. Though probably for a sabbatical, one may guess what kind of impact on British science and etiquette Bert would have left in England had he become established there, which, according to his letter from 1948, he intended to visit the following year. During the 1950s, Bert, in his private dealings, had “…become less disinterested than rather shy, as it is difficult to treat morality in any other way”, from a personal archive addressed by Bert to Karl and Brunhilde in Germany in 1959. For example, he relinquished his claim on the old horse farm in Germany in favour of his family there so that they could pay for education.

## 5. Conclusions

The biography of Bert Vallee provides evidence of the importance of considering a scientist not only as a carrier of scientific output, which in his case filled several filing cabinets in the corridors of the CBBSM. Behind the scientist, there is a real person with private dealings in, driven by, or simply thrown into the circumstances of his time and place. Such an inclusive and rounded approach to Bert Vallee has allowed us to paint a full(er) and, in any case, richer picture of him, which, despite some missing pieces, now allows us to follow him and his scientific career more closely.

Scientists are never sterile carriers of their publications and are often more colourful than one expects or may be ready to accept. In turn, scientists and science should never be considered as entirely “free” of the relevant societal framework, which indeed includes personal and private aspects on the one and wider social, political, economic, and, more recently, environmental considerations on the other side. In fact, such context enhances the edifying nature of biographies. It generates a more lasting legacy of the individual and gives examples of the circumstances for critical decisions in the lives of scientists for the benefit of future generations of aspiring scientists.

Bert had few hobbies and, besides his regular visits to the Harvard and Somerset Clubs and Boston Symphony, mostly kept to himself and to his wife. He also kept a number of secrets which even a skilled biographer could not solve, such as the origin of his middle name Lester, which is notably absent in his letters from the 1940s and then present in the 1950s. Maybe it is a homage to an esteemed colleague he may have met in the U.S., maybe linked to the city of Leicester in the U.K. or his adoration for Red Leicester cheese, or simply a play of words on Lesley College, where his wife was a professor. We may only speculate and keep up the suspense.

Besides his passion for science, Bert kept his passion for horses and horse riding. Additionally, regardless of whether standing at the bench in the laboratory or in the stirrups of his horse at a ranch in Montana, Bert Vallee had always been an optimist. As he writes in his letter from 1948, less than ten years after arriving in the U.S. as an immigrant with barely more than a suitcase, and just a few years after the Second World War, “The perspectives for the next couple of years are excellent”. As the scientists of today, we should listen to this and share his optimism.
